# Gene-modified genotype II live attenuated African swine fever virus induces cross-protection against genotype I but not against genotype IX

**DOI:** 10.1080/22221751.2025.2505645

**Published:** 2025-05-12

**Authors:** Anusyah Rathakrishnan, Johanneke D. Hemmink, Vlad Petrovan, Ana Luisa Reis, Linda K. Dixon

**Affiliations:** a The Pirbright Institute, Woking, United Kingdom; b International Livestock Research Institute, Nairobi, Kenya

**Keywords:** African swine fever virus, vaccine, cross-protection, genotype, immune response, immune evasion

## Abstract

African swine fever virus originated in a wildlife cycle in East Africa and spread to domestic pigs. At least twenty-three genotypes are present in Africa whereas only genotypes I and II have spread to other continents. Vaccine development has been directed mainly to genotype II. The ability of genotype II vaccines to induce cross-protection against other genotypes is unknown. Here, we compared cross-protection induced in pigs by an attenuated multiple gene-deleted genotype II modified live vaccine candidate against challenge with different genotypes. Protection against homologous virulent genotype II virus was 100%. Cross-protection against virulent genotype I virus varied between 57 and 71%. However, no protection was achieved against genotype IX challenge. The results indicate potential for use of vaccines in regions where genotypes I and II are circulating.

## Introduction

African swine fever virus (ASFV) is maintained in wild suids and *Ornithodoros* species of soft ticks in East and Southern Africa causing few clinical signs. In domestic pigs and wild boar, ASFV causes a haemorrhagic disease with fatality approaching 100%.

ASFV is a large cytoplasmic DNA virus coding for approximately 170 to 190 proteins. Partial sequencing of the B646L gene, which codes for the major capsid protein p72, identified at least 23 genotypes in Africa [1]. These originate from the sylvatic cycle in East Africa. However, only five genotypes, I, II, VIII, IX and X, have been reported to have an extended field presence in domestic pigs. Genotype I is the prevalent strain in West and Central Africa and genotypes IX, X and VIII in East African countries [2]. Genotype II was first described in Madagascar and Mozambique but has now been detected in a further 8 African countries including two West African countries [3]. Phylogenetic analysis of complete ASFV genome sequences from 9 genotypes available in the NCBI database identified 6 distinct clusters. Genotypes IX and X clusters are most closely related to each other but most widely separated from the other genome clusters [3]. Genotype II ASFV spread to Georgia in the Caucasus region and from there to Russia and Eastern Europe, entering the European Union in 2014. In 2018, genotype II ASFV spread to China, causing huge economic losses and a large reduction of the pig population. Subsequently, ASFV has spread to most Asian countries, several in Oceania and the Caribbean (FAO Empres, WOAH WAHIS). In 2021, low virulent genotype I viruses [4] and in 2023, a highly virulent hybrid genotype I, genotype II virus was detected in China [5]. This hybrid virus was later detected in Vietnam and Russia [6,7].

Two modified live vaccines (MLV) for genotype II ASFV were licensed for use in Vietnam in 2023 [8-10]. However, the potential for induction of cross-protection by genotype II MLVs against different genotypes has been little studied and this remains a crucial knowledge gap for the effective use of ASFV vaccines globally. The ability of sera from infected pigs to inhibit red blood cell binding to infected cells (HAD), correlated with cross-protection and identified 8 ASFV serotypes. Sequences of the *EP402R* gene coding for the protein required for HAD, pCD2v, and the adjacent *EP153R* gene, were used to assign additional strains in serotype groups [11,12] which may predict cross-protection. However, few of these predictions have been tested experimentally with currently circulating strains. Previously an attenuated genotype I virus (BA71ΔCD2v) was shown to induce cross-protection against genotype II. Cross-protection against virulent genotype IX was achieved after boosting with virulent genotype I but not after boost with the same attenuated genotype I [13]. Similarly, immunization of pigs with natural attenuated genotype I strain OURT88/3 and boost with virulent genotype I OURT88/1 induced cross-protection against genotype X challenge [14].

Here we investigated cross-protection induced by an attenuated multiple gene-deleted genotype II strain [15] against prevalent genotypes I and IX, that circulate in domestic pigs in Africa. High levels of protection against homologous virulent genotype II challenge were induced and partial cross-protection against challenge with virulent genotype I but no protection was obtained following challenge with virulent genotype IX virus. These results suggest that MLV ASFV vaccines based on genotype II may be useful in regions where genotype I is circulating.

## Materials and methods

Full experimental details are provided in Supplementary information.

### Cells and viruses

Porcine bone marrow cells (PBMs) were cultured in EBSS supplemented with 10% pig serum, 1% pen-strep and 1% HEPES. Recombinant ASFV modified live vaccine (MLV) candidate, GeorgiaΔDP148RΔK145RΔEP153R-CD2v_mutantQ96R/K108D (GΔDKE-Cmut) [15], virulent genotype II ASFV Georgia 2007/1, genotype I Benin 97/1 [16,17] and Kenya 1033 genotype IX isolate were described previously [18].

### In vivo immunization and challenge of pigs

**Figure 1.**
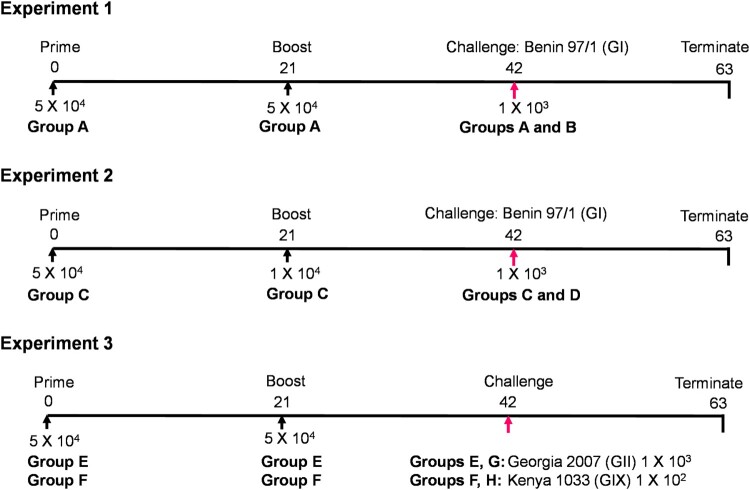
Experimental timelines. In every experiment, the pig group numbers, days, doses and viruses used for immunization, boost and challenge are shown.

All three experiments were carried out in ILRI, Kenya as shown in Figure 1. Groups of 7 pigs were immunized and boosted with GΔDKE-Cmut. Immunized pigs were challenged with virulent virus of genotypes I, II or IX in parallel with groups of 3 or 4 naïve pigs as controls for the inoculum. Daily clinical scores were obtained [14] and blood samples were collected at different days post-immunization (dpi) and days post-challenge (dpc) as well as at termination. Animals were euthanized at a moderate severity humane endpoint or at the end of the study. At necropsy, macroscopic lesions typical of ASFV infection were scored [19].

### ASFV genome in blood

DNA was extracted from whole blood collected in EDTA vacutainers and ASFV genome copies were measured by real time PCR. Standard curves from an ASFV p72 mimic plasmid were included to allow for genome quantification [20]. Results are presented as log_10_ genome copies/mL. The cut-off for accurate detection was 10^3^ ASFV genome copies/mL. The virus genome levels were defined as low if ≤10^4^ genome copies/mL, moderate if between 10^4^ and 10^6^ genome copies/mL and high if ≥10^6^ genome copies per mL.

### ASFV P72 antibodies in serum

Antibody responses against the ASFV p72 protein were measured in serum using a commercial blocking ELISA (INgezim PPA Compac, Ingenasa). Samples showing >50% blocking rate were considered as positive, while anything <40% was considered as negative. Samples with blocking between 40 and 50% were considered as doubtful.

### IFN-γ cellular responses in pigs

Levels of the IFN-γ cellular responses in the isolated PBMCs of vaccinated pigs were measured after stimulation with different virus strains using an ELISpot assay [15]. The results are presented as spot forming cells (SFC) per million PBMC.

### Statistical analyses

Statistical analyses were used to evaluate the differences between immunized and naïve control pigs from day 0 post-challenge (42 dpi) up to 6- or 7 dpc. The parameters compared include rectal temperatures, clinical scores, post-mortem scores and genome copies/mL. Two-way ANOVA either using the mixed effect model (when missing values are present) or repeated measure model, both with Geisser-Greenhouse correction, was carried out alongside Tukey’s multiple comparisons test (GraphPad Prism 10) for all parameters except post-mortem scores. For the post-mortem scores, unpaired, non-parametric, Mann–Whitney test was employed to evaluate differences in the total cumulative scores between groups.

## Results

Pigs were immunized and boosted with an attenuated genotype II virus with deletions of *DP148R*, *K145R*, and *EP153R,* expressing a non-HAD pCD2v protein with mutations of residues Q96R and K108D. The challenge was with virulent genotype I Benin 97/1, virulent genotype II Georgia 2007/1 [15] or virulent genotype IX Kenya 1033 [18]. Figure 1 shows the timeline of each of the experiments.

### Immunization and boost with GΔDKE-Cmut partially protects pigs against challenge with virulent genotype I strain Benin 97/1

#### Clinical scores and temperatures

**Figure 2.**
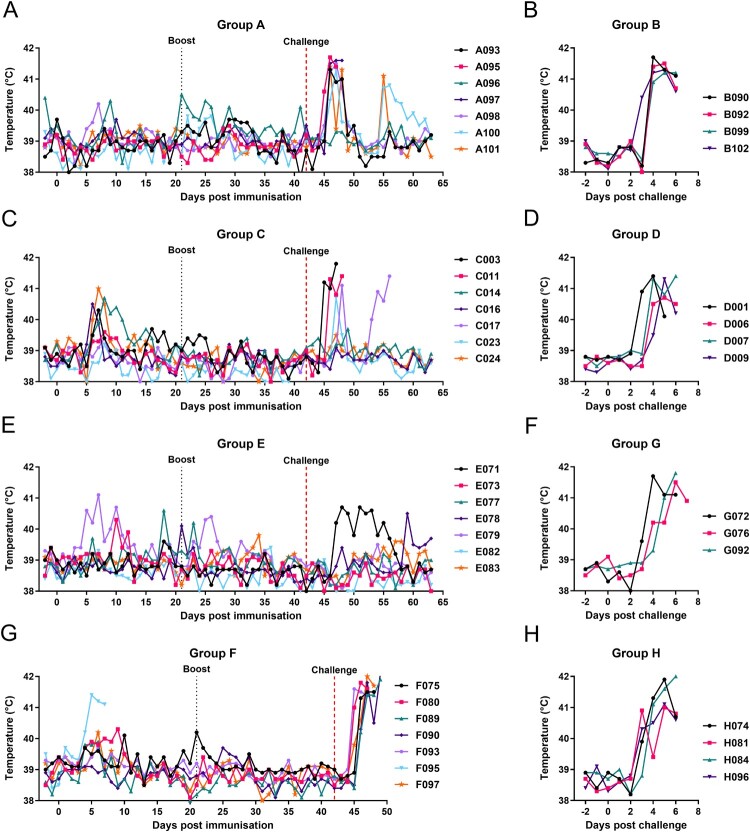
Daily temperatures of pigs. Daily rectal temperatures for pigs in each of the three experiments are shown. Panels A and B show results from experiment 1, panels C and D from experiment 2, panels E, F, G, and H from experiment 3.

**Figure 3.**
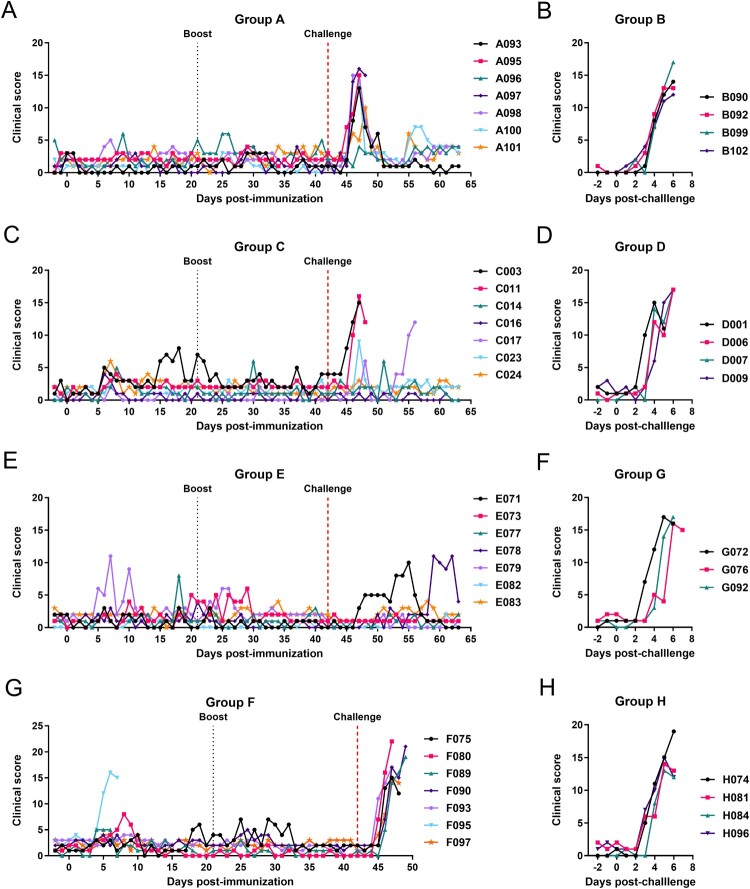
Clinical scores of pigs. Panels A and B show results from experiment 1, panels C and D from experiment 2, panels E, F, G and H from experiment 3.

In the first experiment, one group of 7 pigs (Group A) was immunized intramuscularly with 5 × 10^4^ TCID_50_ of GΔDKE-Cmut MLV and boosted by the same route and dose at 21 dpi. At 42 dpi, the pigs were challenged intramuscularly with 10^3^ HAD_50_ genotype I Benin 97/1 in parallel with 4 naïve pigs (Group B). After immunization and boost, none of the animals in Group A developed a body temperature above 40.5°C. Mild transient clinical signs including lethargy or joint swelling were observed in some animals. These are likely to have been non-procedure related and were also observed in some pigs before immunization (Figures 2 and 3(A)).

After challenge, all pigs in Group A showed an increase in temperature above 40.5°C. Two pigs reached the moderate severity humane endpoint (A095 and A097) on 47 or 48 dpi (5- or 6 dpc). The remaining five pigs survived until the end of the experiment and had temperatures above 40.5°C for one to three days. Maximum daily clinical scores after challenge in Group A, were between 13 and 16 on 46 or 47 dpi for five pigs, including those that reached the humane endpoint on 47 or 48 dpi. The other two pigs (A096 and A101) had a maximum daily clinical score of 4 or 10 on 47 and 48 dpi, respectively. Daily clinical scores reduced after 48 dpi in the pigs that survived until the end of the experiment.

All the control pigs (Group B), challenged with genotype I, reached the humane endpoint by 6 dpc. Temperatures increased to above 40.5°C from 3 dpc (Figure 2(B)). Other clinical signs included loss of appetite and reluctance to get up (Figure 3(B)).

The mean temperatures for pigs in Groups A and B after challenge were similar with no statistical difference except on the day of challenge when pigs in Group A had statistically higher mean temperatures than those in Group B (Figure S1A). Comparisons of the mean clinical scores between pigs in Groups A and B showed similarly increasing scores between 3 to 6 dpc. At 6 dpc, mean scores for pigs in Group A were statistically lower than for those in Group B (*p* = 0.0197) (Figure S2A).

#### ASFV genome in blood

**Figure 4.**
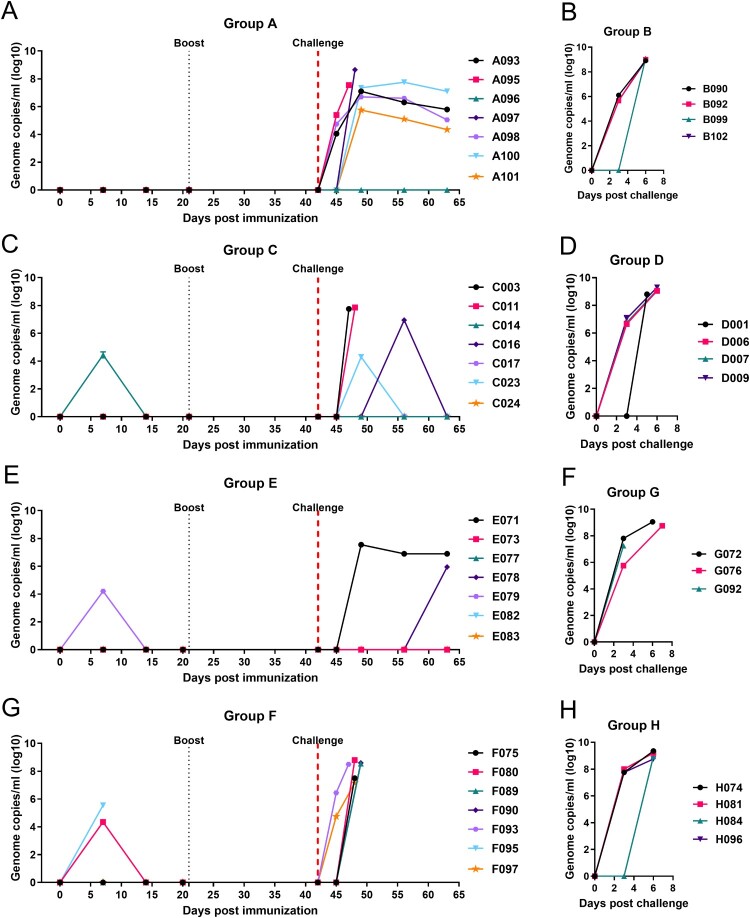
Virus genome copies in blood. Virus genome copies per mL EDTA blood were measured by qPCR in samples collected at the indicated time points in each experiment. Values are shown on the y-axis on a log 10 scale. Panels A and B show results from experiment 1, panels C and D from experiment 2, panels E, F, G and H from experiment 3.

No virus genome was detected post-immunization or boost in any of the pigs in Group A (Figure 4(A)). After challenge with genotype I Benin97/1 at 42 dpi, the two Group A pigs which reached the humane endpoint on 47 or 48 dpi, had 10^7.6^ or 10^8.7^ genome copies/mL blood. In the pigs that survived challenge, virus genome was detected between 49 to 63 dpi, decreasing from the highest values of 10^5.7-7.8^ on 49 dpi to 10^4.4-7.0^ at termination (63 dpi). One pig, A096, had no virus genome detected in blood after challenge (Figure 4(A)).

In the challenge control Group B, virus genome in blood was detected at 3 dpc in three of the four pigs. On the day of cull, these pigs had genome copies per mL of 10^8.9-9.1^ (Figure 4(B)).

Mean values for virus genome copies per/mL were lower in pigs in Group A compared to Group B at 3 dpc reaching statistical significance (*p* = 0.0399) at the peak of viremia measured on 5-, 6- or 7 dpc (Figure S3A).

#### Macroscopic lesions observed at necropsy

**Figure 5.**
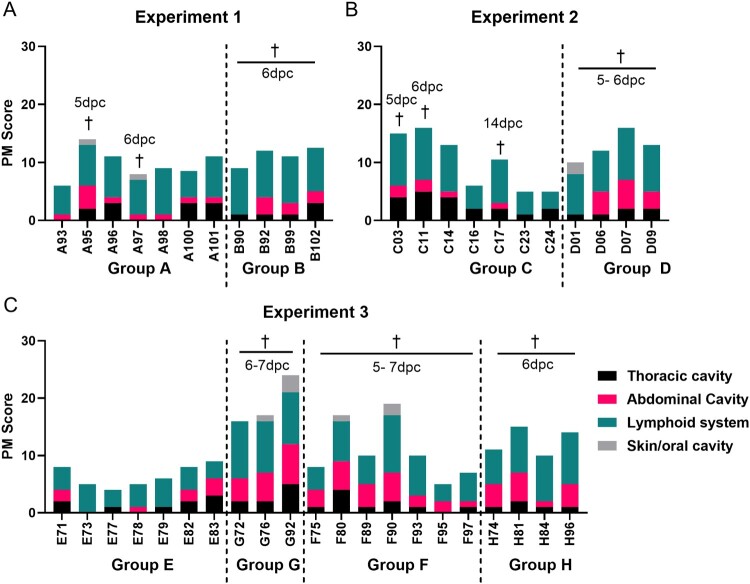
Macroscopic lesion scores at termination. Macroscopic lesions were scored at termination either at a moderate severity humane endpoint or at the end of the experiment for surviving pigs. Panels show results from experiments 1 to 3. The x-axis shows the pig number and y axis the cumulative lesion score. Colours indicate lesions observed in the thoracic, abdominal, and skin/oral cavities as well as in the lymphoid system. Lesions in the thoracic cavity include the presence of thoracic exudates as well as lesions affecting cardio-respiratory system (black bar). Lesions in the abdominal cavity include the presence of ascites along with the presence of lesions affecting the gastrointestinal system including the stomach, intestines, liver and gallbladder (pink bar). Lesions in lymphoid tissues include tonsils, thymus, spleen and several lymph nodes (teal bar). The grey bar shows lesions in skin and oral cavity.

One pig from Group A (A095), which reached the humane endpoint after challenge, had higher lesion scores compared to the second pig which reached the endpoint (A097) (Figure 5(A)). Lesions observed included enlargement and signs of haemorrhage in the spleen and several lymph nodes. Other signs included petechias on the medulla of the kidney, mild lung oedema, pericarditis and ascites. Three pigs (A096, A100, A101) had severe pericarditis and one pig (A095) had mild pericarditis. The mucosa of stomach and duodenum were slightly haemorrhagic in 4 pigs and one pig had skin haemorrhages in the ears. In control Group B, lesion scores associated with acute ASF ranged between 9 and 12.5 (Figure 5(A)). When compared, no significant differences in lesion scores were observed between individual pigs nor between groups A and B (Figure S4A).

#### Immune responses

**Figure 6.**
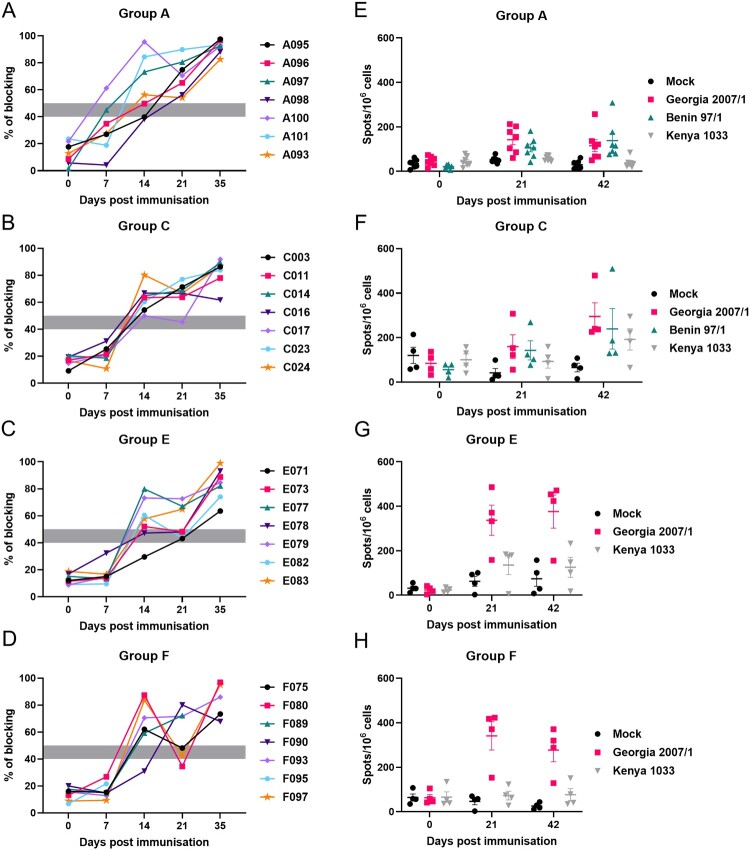
Antibody and cellular immune responses. Antibodies against ASFV p72 were measured using a commercial blocking ELISA. The results from different pig groups as labelled are shown in panels A, B, C and D. The x-axis shows days post-immunization that samples were collected. The y-axis shows % blocking. PBMCs collected before immunization, boost and challenge of pigs from Groups A, C, E and F were either mock-stimulated or stimulated with genotype II Georgia 2007/1, genotype I Benin97/1 or genotype IX Kenya 1033. Panels E, F, G and H show numbers of IFN-γ producing cells per 10^6^ cells measured by ELISpot assay (y-axis) and days post-immunization that cells were collected are shown on the x-axis.

Serum levels of ASFV-specific antibodies (Figure 6(A)) were above the cut-off in 4 pigs in Group A at 14 dpi and in all animals by 21 dpi.

The numbers of IFN-γ producing cells in pigs from Group A showed a trend to be higher after genotype II stimulation, followed by genotype I and lowest for genotype IX stimulation (Figure 6(E), S5A-D). However, these differences were not significant and did not correlate with survival of individual pigs.

### Immunization and boost with a lower dose of attenuated genotype II GΔDKE-Cmut partially protects pigs from challenge with virulent genotype I Benin 97/1

#### Temperatures and clinical scores

In experiment 2, we used the same prime dose as in experiment 1 but reduced the boost dose by 5-fold to determine if this may affect the levels of protection. Seven pigs (Group C) were immunized with GΔDKE-Cmut at 5 × 10^4^ TCID_50_ and boosted with 1 × 10 ^4^ after 21 days. After a further 21 days, the pigs were challenged with Benin 97/1 10^3^ HAD_50_ in parallel with a control group of 4 naïve pigs (Group D) (Figure 1). After immunization, pig C024 had a temperature of 41°C at 7 dpi and pig C014 had a temperature of 40.7°C at 8 dpi. No further temperatures above 40.5°C were detected post-immunization or boost (Figure 2(C)). Mild transient clinical signs including lethargy or joint swelling were observed in some animals (Figure 3(C)). These are likely to have been non-procedure related and were also observed in some pigs before immunization.

After challenge at 42 dpi, three pigs (C003, C011 and C017) reached the moderate severity endpoint at 47-, 48-, and 56 dpi, respectively (5-, 6- or 14 dpc, respectively). Of the four pigs which survived, one had a temperature >40.5°C on 47 dpi (5 dpc) but no further temperatures >40.5°C or other clinical signs. The other three Group C pigs had no increase in temperatures (Figures 2 and 3(C)).

The 4 pigs in the non-immune control Group D were all terminated on 5 or 6dpc as expected, showing signs typical of acute ASF including high temperatures, loss of appetite and increasing lethargy (Figures 2 and 3(D)).

No significant mean differences in temperature were detected between pigs in Groups C and D after challenge (Figure S1B). However, when the overall clinical scores were compared between Groups C and D, the scores of the immunized pigs were clearly lower than the naïve-challenged pigs, with significant difference on 4 dpc (*p* = 0.022) and on 6 dpc (*p* = 0.0008) (Figure S2B).

When we compared Group A (from Experiment 1) which received the same immunization dose as Group C, but a 5-fold higher booster dose, no significant differences were found after challenge in clinical scores (Figure S2C). The only significant difference was noted for temperatures on 4 dpc (*p* = 0.0287) (Figure S1C).

#### Virus genome in blood

One pig in Group C (C014) had a moderate level, 10^4.5^, of genome copies per mL blood on 7 dpi. No virus genome was detected in any of the other Group C pigs after immunization or boost (Figure 4(C)).

After challenge, high genome copy numbers per mL (10^7.75-7.85^) were detected in blood from two of the pigs (C003 and C011), which were euthanized at the humane end point on 5 or 6 dpc, respectively. Surprisingly, no virus genome was detected in blood from the other pig which was culled on 14 dpc at the humane endpoint (Figure 4(C)). At 7 dpc, ASFV genome copies of 10^4.3^ per mL were detected in blood from pig C023, and at 14 dpc 10^6.9^ genome copies per mL blood in pig C016. In the other surviving pigs, no virus genome was detected after challenge. As expected, high levels of genome copies per mL (10^8.8-9.3^) were detected in blood from pigs in control Group D at termination (Figure 4(D)). When compared, Group C showed lower levels of virus genome copies per mL blood than Group D, and at 5, 6 or 7 dpc, these differences were statistically significant (*p* = 0.0045) (Figure S3B).

The mean genome copies per mL blood for pigs in Group C tended to be lower in comparison with mean values for pigs in Group A although they were not statistically significant (Figure S3C).

#### Macroscopic lesion scores at necropsy

At necropsy, the three pigs from Group C (C003, C011 and C017), which reached the humane endpoint, showed enlargement and signs of haemorrhage in the spleen and several lymph nodes. Mild or moderate lung oedema and pericarditis were observed in these three pigs and one pig had dark kidneys with petechias and mild haemorrhagic mucosa in the stomach and duodenum (Figure 5(B)). Of the pigs that survived until termination of the experiment, one pig (C014) had lesions typical of ASF. The other surviving pigs had, as expected, fewer lesions and low lesion scores of 5 or 6.

The control pigs in Group D had more severe signs typical of acute ASF (Figure 5(B)). Comparisons of the total scores showed no statistical significance between the pigs in Group C and D (Figure S4B). We also found no significant differences between immunized and genotype I challenged pigs of Group A and C (Figure S4C).

#### Immune responses

Antibody levels above the cut-off were detected in five pigs in Group C by 14 dpi and in all animals by 35 dpi (Figure 6(B)).

The highest numbers of IFN-γ producing cells were seen in response to genotype II virus stimulation, which increased after boost in most pigs. On average, responses to genotype I and IX were lower but some outliers showed a higher response (Figure 6(F), S5E-H). Interestingly, pig C011, which reached the humane end point at 6 dpc, had the highest number of IFN-γ producing cells following stimulation with both Georgia 2007/1 and Benin 97/1 (Figure S5E-H).

### Immunization and boost with GΔDKE-Cmut induces protection against virulent genotype II challenge but no cross-protection against virulent genotype IX challenge

The ability of the attenuated genotype II virus GΔDKE-Cmut to induce cross-protection against virulent genotype IX Kenya 1033 was compared with protection induced against genotype II virus challenge. Two groups of seven pigs (Groups E and F) were immunized intramuscularly with 5 × 10^4^ TCID_50_ of GΔDKE-Cmut on day 0 and pigs were boosted at 21 dpi with the same dose and route. At 42 dpi, pigs in Group E were challenged with 10^3^ HAD_50_ Georgia2007/1 genotype II in parallel with three naïve pigs as controls for the challenge (Group G). Immunized pigs in Group F were challenged with 10^2^ HAD_50_ Kenya 1033 genotype IX in parallel with four challenge control pigs (Group H) (Figure 1).

#### Clinical scores and temperatures

After immunization, one pig in Group E, E079, had temperatures above 40.5°C for 3 separate days between 5- to 10 dpi and another pig (E077) for one day, at 18 dpi (Figure 2(E)). After challenge, at 42 dpi, with genotype II Georgia 2007/1, one pig (E071) had a temperature >40.5°C on three separate days, 48-, 51- and 53 dpi (6, 9 and 11 dpc). No other pigs had temperatures above 40.5°C and all pigs survived until the end of the experiment (Figures 2 and 3(E)).

In the other immunized group (Group F), one pig (F095) had a temperature above 41°C for three days post-immunization (5–7 dpi) accompanied by other signs including lethargy and two days not eating and was terminated at the humane endpoint at 7 dpi (Figures 2 and 3(G)). None of the other pigs had temperatures above 40.5°C after immunization or boost. After challenge with Genotype IX Kenya 1033, all the pigs in Group F had 3 days of temperature above 41°C, lethargy and were not eating for two days. They were terminated at the humane end point on 5 to 7 dpc (Figures 2 and 3(G)). Mild transient clinical signs post immunization, including lethargy or joint swelling, were observed in some animals in Groups E and F. These are likely to have been non-procedure related and were also observed in some pigs before immunization.

The control groups challenged with genotype II Georgia 2007/1 (Group G) or Kenya 1033 (Group H) developed clinical signs of acute ASF including temperatures of 40.5°C to >41.0°C for 3 days and other clinical signs including lethargy and two days of not eating, and were terminated on 6 or 7 dpc (Group G) or 6 dpc (Group H) at the humane endpoint (Figures 2, 3(F,H)).

Significant differences were found in the both the temperatures and clinical scores between Group E (immunized) and G (naïve), after challenge with the homologous virulent Georgia 2007/1 strain. Temperatures for the immunized Group E pigs were significantly lower than Group G on 6 (*p* = 0.022) and 7 dpc (*p* = 0.0008) (Figure S1D), while for the overall observed clinical signs, they were significantly lower at 2 dpc (*p* = 0.03) and 6 dpc (*p* = <0.0001) (Figure S2D). In contrast, temperatures and clinical scores for pigs in Group F (immunized) and control Group H showed no statistical differences (Figures S1E and S2E).

#### Virus genome in blood

After immunization, only one pig in Group E had detectable genome in blood, 10^4.2^ genome copies per mL at 7 dpi (Figure 4(E)). In Group F, pig F095, which reached its humane endpoint on 7 dpi had genome copies of 10^5.5^ per mL blood. This moderate level of virus genome in blood suggests other factors may have contributed to this pig reaching the humane endpoint. One other pig in Group F, F080, had 10^4.4^ genome copies per mL on 7 dpi (Figure 4(G)).

After challenge with virulent genotype II, two pigs in Group E had virus genome detected in blood. The levels ranged in one pig (E071), from 10^7.5^ on 49 dpi to 10^6.9^ genome copies per mL at termination. The other pig, E079, had 10^5.9^ genome copies per mL at termination. No other pigs in Group E had detectable virus genome in blood (Figure 4(E)).

The 6 pigs in Group F, challenged with genotype IX, were terminated between 5 and 7 dpc (47–49 dpi). Genome copies per mL of blood detected at termination were 10^8.5^ or higher in most pigs (Figure 4(G)). Pigs F075 and F097 had genome copies per mL blood of 10^7.5^ and 10^7.1^ at termination, respectively.

In control Group G, challenged with virulent genotype II Georgia 2007/1, pigs had genome copies per mL of 10^7.2-9.1^ at the humane endpoint (Figure 4(F)). In control Group H, challenged with virulent genotype IX, all four pigs had genome copies per mL between 10^8.9-9.1^ at termination at the humane endpoint on 5 or 6 dpc (Figure 4(H)).

Comparison of mean values for genome copies/mL in blood between immunized pigs in Group E and control pigs in Group G showed significantly lower virus genomes in Group E at both 3 dpc (*p* = 0.0075) and 5 to 7 dpc (*p* = 0.0003) (Figure S3D). Despite reaching their humane endpoints, immunized pigs in Group F, that were challenged with genotype IX virulent isolate, had statistically significant lower virus genome copies per mL blood compared to the control Group H (*p* = 0.0249) (Figure S1E). This indicates that the immunization and boost with GΔDKE-Cmut virus had induced an immune response which reduced replication of the challenge virus, but not enough to afford protection.

#### Macroscopic lesion scores at necropsy

Pigs in Group E, immunized and challenged with genotype II, all had mild signs of haemorrhage detected in lymph nodes (Figure 5(C)). Four pigs had signs of haemorrhage in the duodenum, one had small petechias on both kidneys, five pigs had mild lung oedema, and five pigs showed signs of pericarditis. The three pigs in control Group G, challenged with virulent genotype II, had more severe lesions mostly in the lymphoid system. One pig had petechias through the cortex of both kidneys, and all three had haemorrhagic mucosa in the duodenum. All had lung oedema ranging from mild to severe. When the total lesion scores were compared between Group E and Group G, the immunized, protected pigs had significantly lower scores than the non-immune pigs (*p* = 0.0167) (Figure S4D).

In Group F, all immunized pigs challenged with genotype IX ASFV, had haemorrhagic lymph nodes and enlarged haemorrhagic spleens. In addition, three pigs had small petechias in the kidneys. Five pigs had signs of haemorrhage in mucosa of the stomach and duodenum and five had mild lung oedema (Figure 5(C)). The four naïve pigs in Group H, all had haemorrhagic lymph nodes and spleens. Small petechias were detected in kidneys in two pigs. All four pigs had signs of haemorrhage in mucosa of the duodenum and mild lung oedema. As expected, no significant differences were observed between the immunized pigs and naïve control pigs after challenge (Figure S4E).

In conclusion, there was no evidence that the attenuated genotype II GΔDKE-Cmut protected pigs against challenge with virulent genotype IX. All pigs immunized with the same vaccine and challenged with virulent genotype II survived.

#### Immune responses

Antibody responses in 10 pigs were above the cut-off at 14 dpi, and good levels were observed in all animals by 35 dpi (Figure 6(C,D)).

IFN-γ cellular responses to genotype II stimulation were highest in both Groups E and F and increased after boost in most pigs. Responses to genotype IX stimulation were much lower (Figure 6(G,H) and S5I-N).

## Discussion

Control of ASFV is hindered by several factors including limited availability of vaccines [21]. Modified live vaccines provide the most promising approach, but their use is limited by a lack of knowledge on the extent of cross-protection that can be achieved between circulating isolates. MLVs based on the genotype II current pandemic strains have been licensed for use in Vietnam. However, these do not induce cross-protection against virulent genotype II and genotype I hybrids which were first detected in China, and subsequently in Vietnam and Russia [5,22]. Cross-protection induced by genotype II MLVs against other genotypes, including prevalent genotypes circulating in domestic pigs in Africa, genotypes I and IX, have not previously been reported.

Cross-protection cannot currently be predicted accurately based on genome sequences or analysis of immune responses *ex vivo*. Antibodies alone are not effective in providing protection and therefore, assays such as *in vitro* neutralization assays, cannot be used to predict cross-protection. Cellular immunity is required for protection induced by attenuated ASFV [23]. Assays based on measuring cellular responses therefore have potential to predict cross-protection. We compared numbers of IFN-γ producing cells detected by ELISpot assay following stimulation of PBMCs, from genotype II vaccinated pigs, with genotype II, I and IX ASFV isolates. This showed a trend of higher values following stimulation with the homologous genotype II, followed by genotype I but without statistical significance. Possibly, screening for responses from specific cellular subsets might show greater correlation with induction of protection.

We show here that our previously described multiple gene-deleted genotype II MLV GΔDKE-Cmut induced partial cross-protection against virulent genotype I challenge but no protection against virulent genotype IX. In the experiments described here, groups of pigs were immunized and boosted with GΔDKE-Cmut and challenged with either virulent genotype I Benin 97/1, genotype II Georgia 2007/1 and genotype IX Kenya 1033.

Pigs were immunized by the intramuscular route with 5 × 10^4^ TCID_50_ GΔDKE-Cmut and boosted with either 5 × 10^4^ or 1 × 10^4^ TCID_50_ GΔDKE-Cmut, leading to survival of 5 out of 7 pigs (Group A) and 4 pigs from 7 (Group C) after challenge with genotype I, respectively. Based on these results it is not possible to draw conclusions about the effect of the booster dose on protection levels. Although the numbers of pigs that survived the challenge beyond the humane endpoint were higher in Group A, this is not statistically significant. In addition, the clinical scores, temperatures and levels of virus genome in blood showed a trend to be higher after challenge in pigs in Group A compared to Group C. Macroscopic lesions observed at necropsy were not significantly different between pigs in the vaccinated Groups A and C and control Groups B and D. Others have noted that macroscopic lesion scores at necropsy do not necessarily correlate with the severity of clinical signs suggesting that other factors are important [24]. Possibly using a more severe humane endpoint may have resulted in a greater difference in lesion scores between the naïve and the immunized pigs after challenge with virulent virus. These results show that partial cross-protection of pigs can be achieved by immunization with genotype II MLV followed by challenge with virulent genotype I. Further optimization of the vaccination regime and challenge route may achieve higher levels of protection. It would be interesting to test if cross-protection could be induced by the genotype II MLV GΔDKE-Cmut against challenge with the virulent genotype I/II hybrid circulating in Asia.

All pigs vaccinated with GΔDKE-Cmut and challenged with genotype IX (Group F) reached the humane endpoint criteria at similar times post-challenge as the challenge control pigs, whereas all the immunized pigs challenged with genotype II (Group E) survived until the end of the study with few clinical signs. In previous studies using this MLV*,* we obtained 83% protection against genotype II challenge using a lower immunization and boost dose of 1 × 10^4^ TCID_50_ and 100% protection using 5 × 10^4^ dose with few clinical signs observed either post-immunization or post-challenge [15]. Even though all animals challenged with genotype IX reached the humane endpoint criteria, a significant difference was observed in levels of virus genome in blood of pigs in Group F compared to control Group H after challenge. This indicates that an immune response had been induced which partially controlled virus replication.

Previous studies showed cross-protection against genotype IX or X ASFV can be achieved by immunization of pigs with attenuated genotype I viruses and boosting with a virulent genotype I virus [13,14]. This suggests that cross-protection between genotypes may be extended by applying a stronger booster dose of a less attenuated strain or possibly a higher or more frequent dose of the same strain used for immunization. The close similarity between available whole genome sequences for genotype I, suggest that the genotype II MLV used in this study may partially protect pigs against a wide range of genotype I isolates.

Most ASFV vaccine research is directed at developing vaccines against genotype II, thus, developing strategies to use these in Africa would have advantages. However, the results of this study, suggest that cross-protection against strains which are genetically more distant from genotype II is unlikely, as there was no evidence of cross-protection against challenge with virulent genotype IX. Complete genome sequencing showed genotype IX isolates are more distantly related to genotype II than genotype I isolates [3] so it is not unexpected that protection against genotype IX was not achieved.

Thus for the control of ASFV in Africa, the development of ASFV vaccines based on the genotypes present in the different regions, such as genotype IX and closely related genotype X circulating in domestic pigs in East Africa, remains important [25,26]. A thorough assessment of the ASFV strains present in a particular area before deployment of MLVs, is therefore crucial to avoid vaccine failure.

The use of *in vivo* experiments to test cross-protective capability is expensive, time consuming and requires the use of contained animal facilities. There is an urgent need to develop methods to avoid the need for *in vivo* challenge experiments. Reagents generated in our experiments will be useful to help establish *ex vivo* assays that can predict cross-protection. Increased knowledge of protective antigens will provide information to help predict cross-protection from genome sequences.

## Supplementary Material

Figure S4.tif

Supplementary_Data_formatted_for_EMI_revised-clean.docx

Figure S2.tif

Figure S3.tif

Figure S6.tif

Figure S1.tif

Figure S5.tif
