# Supplementary figures and images for: Gene-modified genotype II live attenuated African swine fever virus induces cross-protection against genotype I but not against genotype IX
Source: Emerg Microbes Infect. 2025 May 12;14(1):2505645. doi: 10.1080/22221751.2025.2505645 (PMC12093800; doi:10.1080/22221751.2025.2505645)

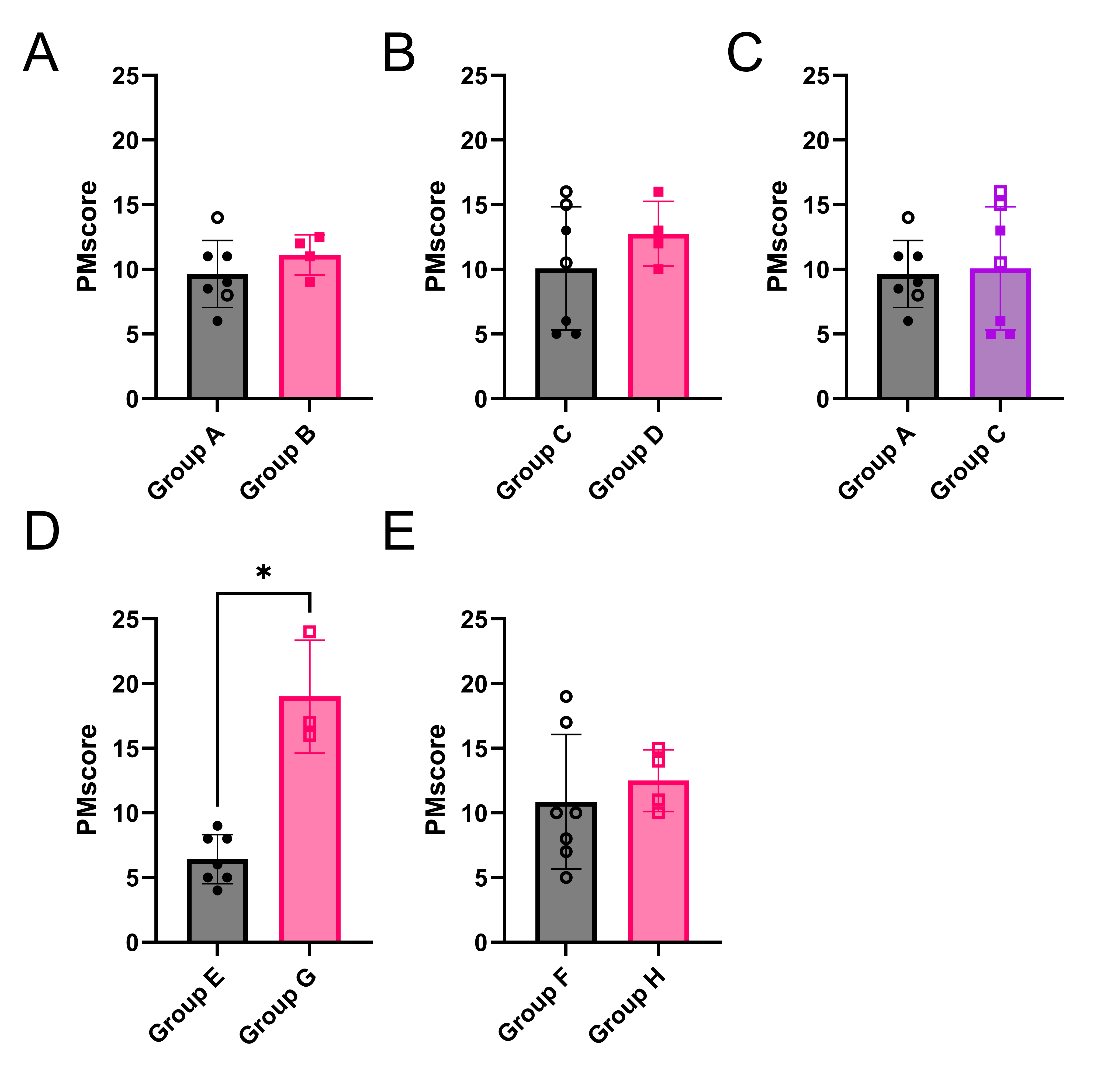

Supplement: Figure S4.tif [file TEMI_A_2505645_SM3508.tif]

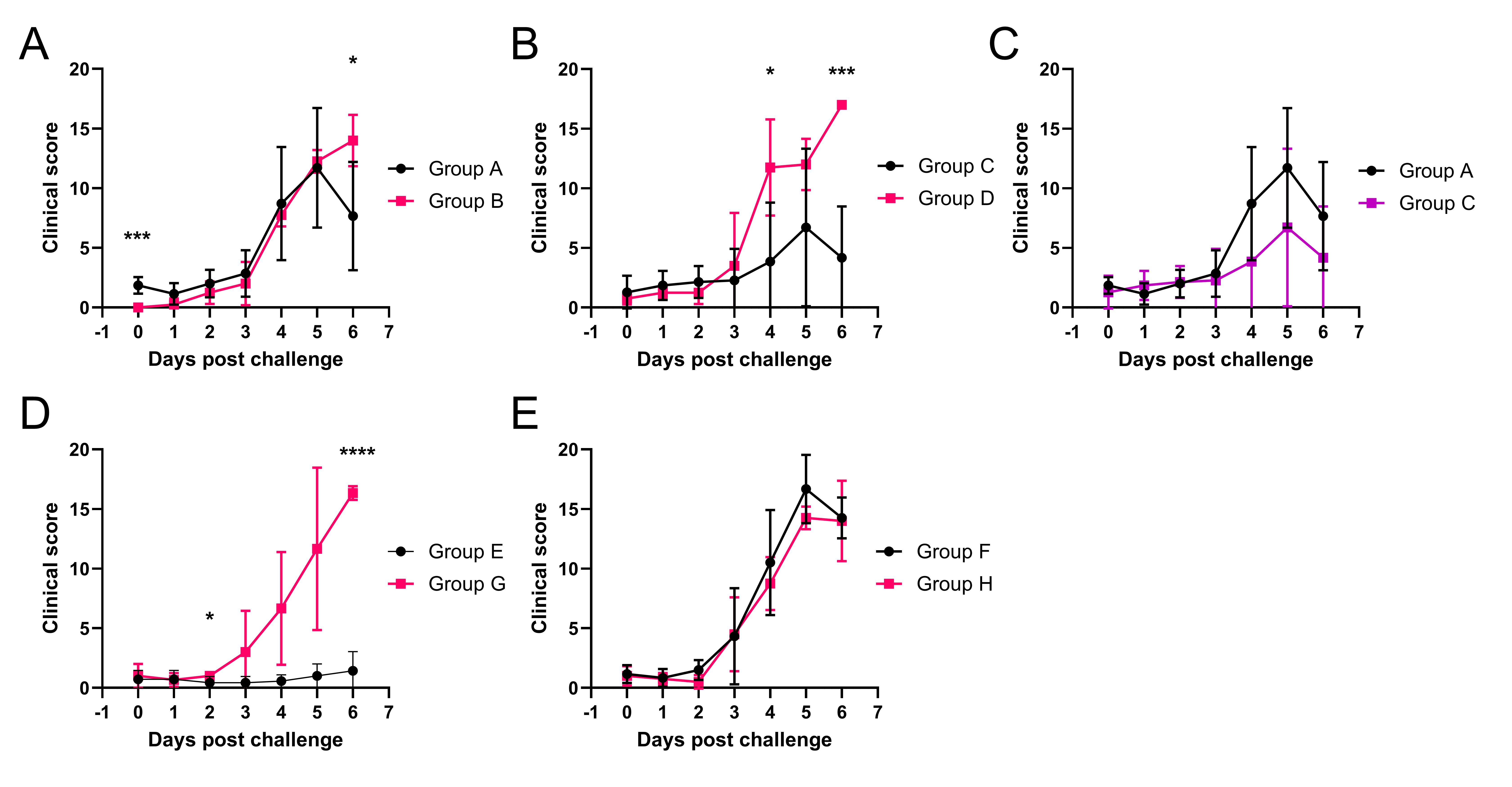

Supplement: Figure S2.tif [file TEMI_A_2505645_SM3506.tif]

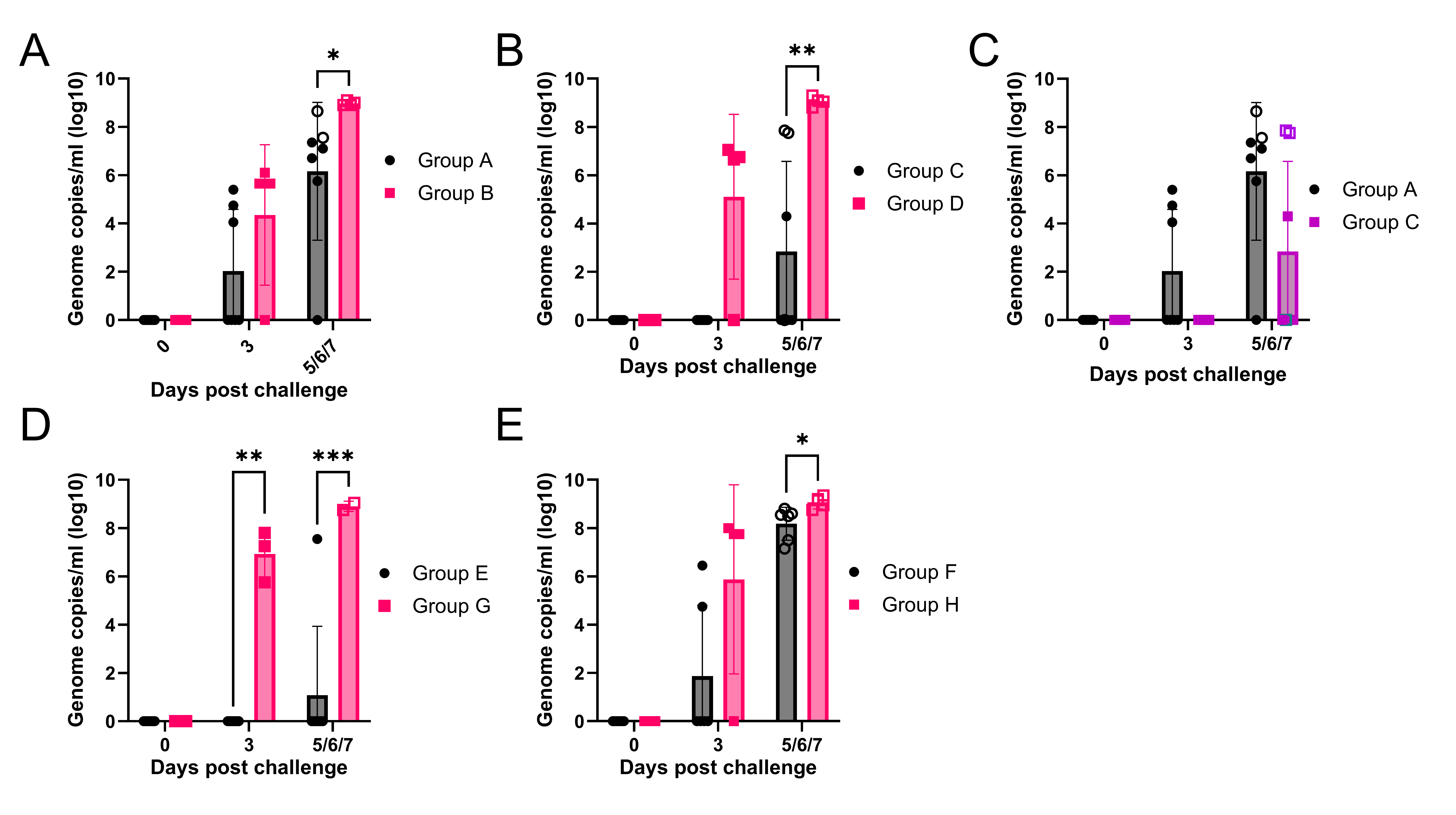

Supplement: Figure S3.tif [file TEMI_A_2505645_SM3505.tif]

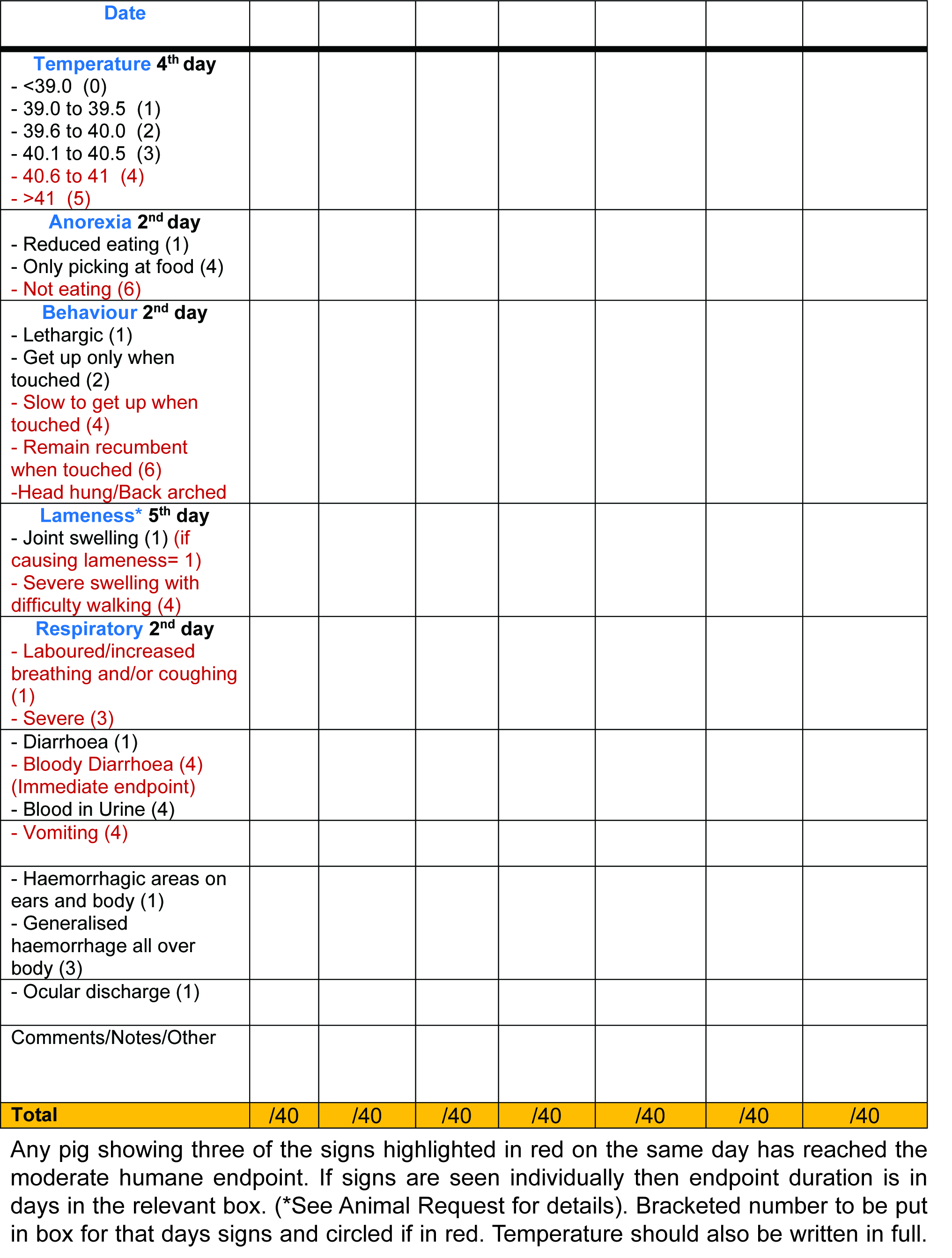

Supplement: Figure S6.tif [file TEMI_A_2505645_SM3504.tif]

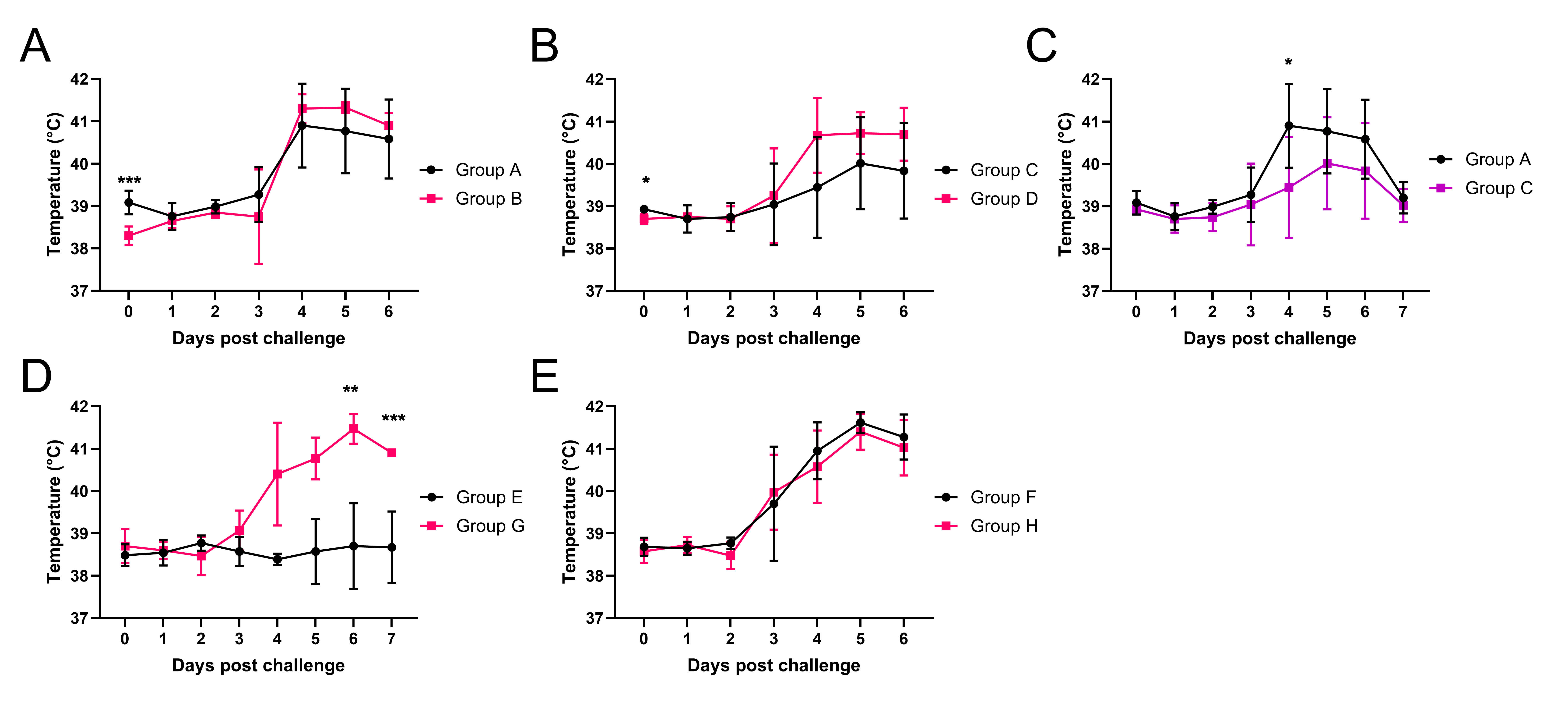

Supplement: Figure S1.tif [file TEMI_A_2505645_SM3503.tif]

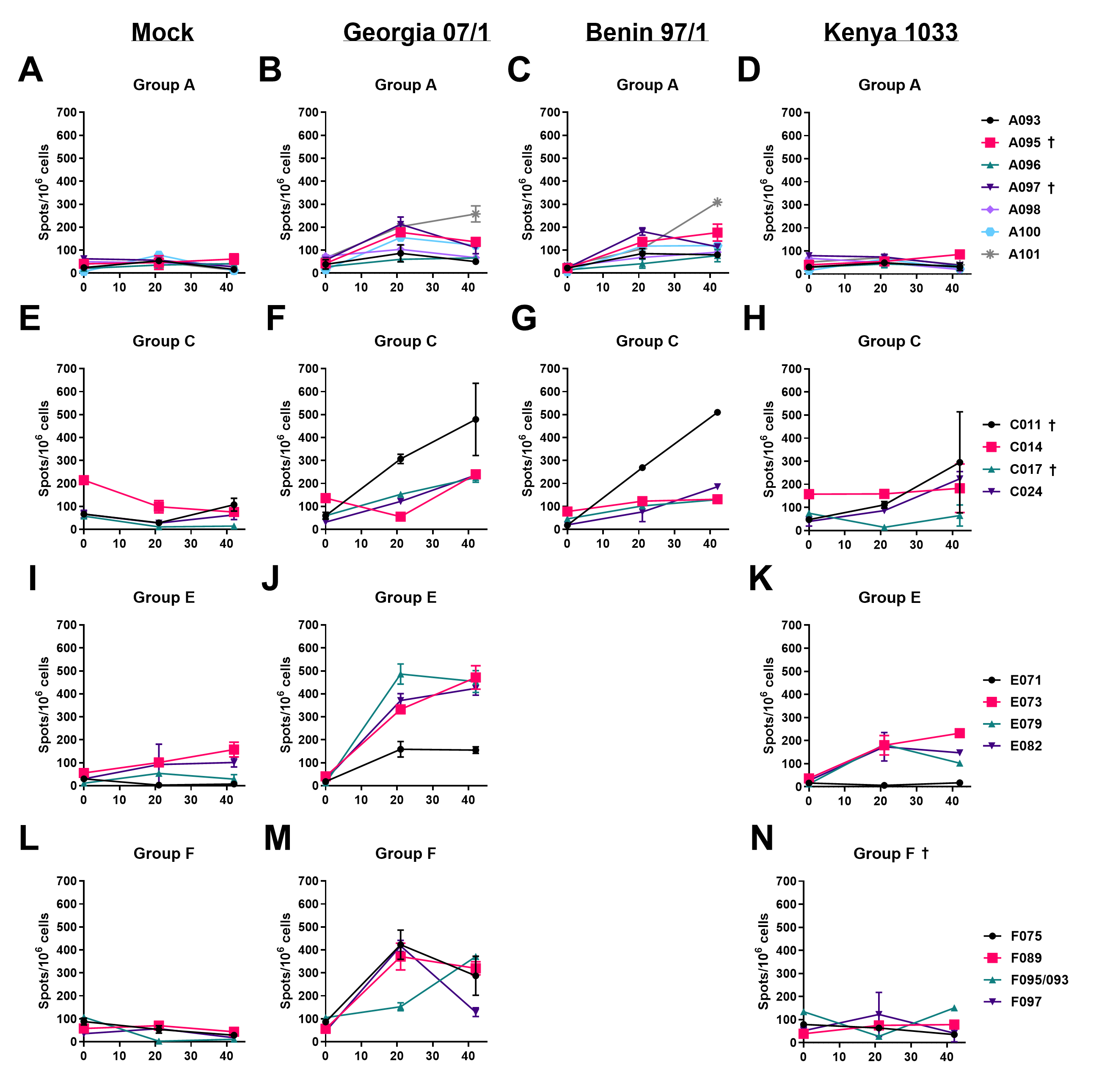

Supplement: Figure S5.tif [file TEMI_A_2505645_SM3502.tif]
