# Supplementary material for: Gene-modified genotype II live attenuated African swine fever virus induces cross-protection against genotype I but not against genotype IX
Source: Emerg Microbes Infect. 2025 May 12;14(1):2505645. doi: 10.1080/22221751.2025.2505645 (PMC12093800; doi:10.1080/22221751.2025.2505645)
Supplement: Supplementary_Data_formatted_for_EMI_revised-clean.docx [file TEMI_A_2505645_SM3507.docx]

# Supplementary data

Gene-modified genotype II live attenuated African swine fever virus induces cross-protection against genotype I but not against genotype IX

Anusyah Rathakrishnan^a^, Johanneke D Hemmink^b^, Vlad Petrovan^a^, Ana Luisa Reis^a^, Linda K Dixon^a^ *

# Supplementary Figures


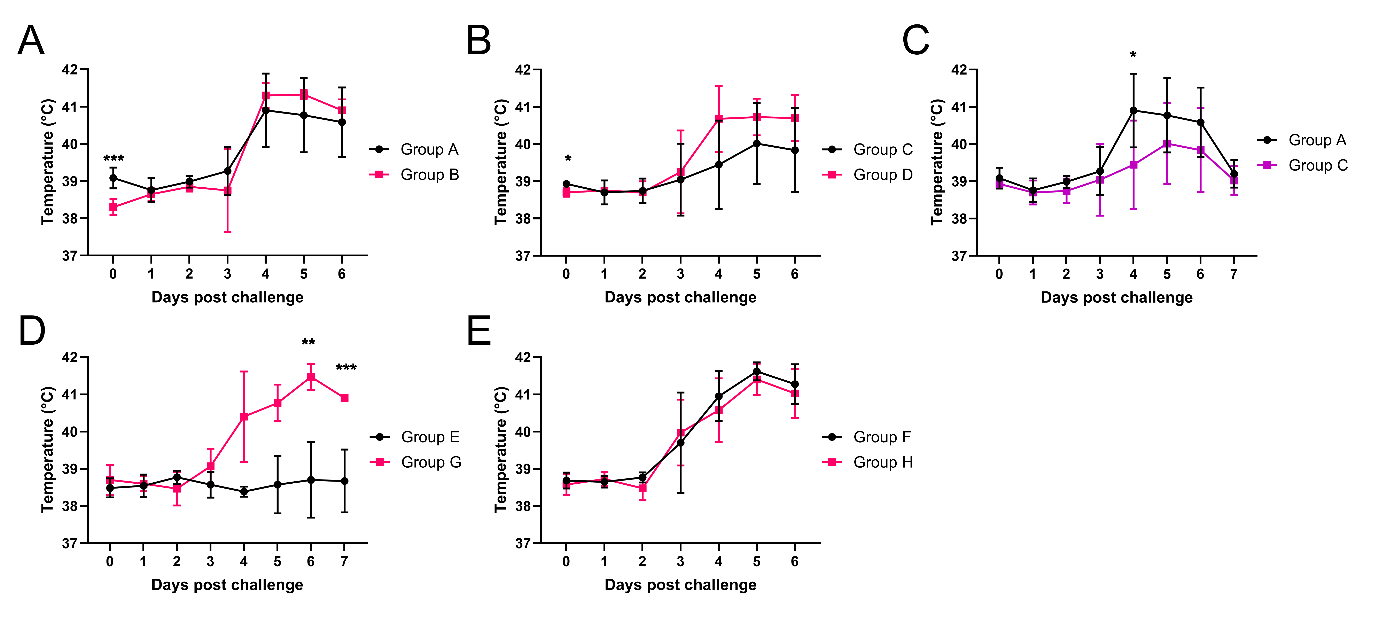


**Figure S1. Comparison of temperatures post-challenge.** Rectal temperatures for pigs in (**A**) Groups A and B, (**B**) Groups C and D, (**D**) Groups E and G, as well as (**E**) Groups F and H, are shown from 42 days post-immunisation (dpi) (0 days post-challenge (dpc)) up to 6 or 7 dpc. Two-way ANOVA using the mixed-effect model with Geisser-Greenhouse correction, and Tukey’s multiple comparisons test was performed to evaluate the differences between vaccinated-challenged and naïve-challenged groups over 6- or 7-days post-challenge. The same statistical analysis was used to evaluate if there was a difference between the Benin 97/1 challenged pigs in Group A and Group C (**C**). * represents P-values, where * <0.05, ** <0.01 and *** <0.001.


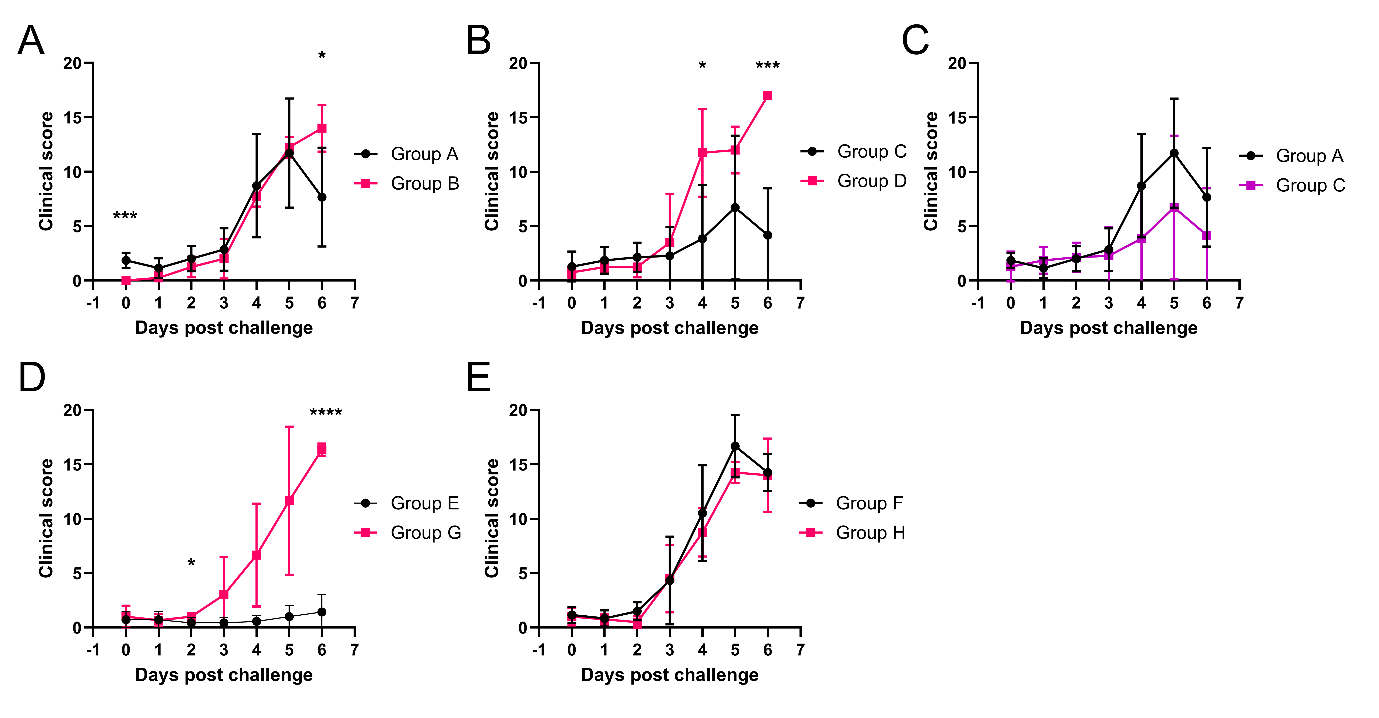


**Figure S2. Comparison of clinical scores post-challenge.** Cumulative clinical scores for pigs in (**A**) Groups A and B, (**B**) Groups C and D, (**D**) Groups E and G, as well as (**E**) Groups F and H, are shown from 42 dpi (0 dpc) up to 6 dpc. Two-way ANOVA using the mixed-effect model with Geisser-Greenhouse correction, and Tukey’s multiple comparisons test was performed to evaluate the differences between vaccinated-challenged and naïve-challenged groups over 6 days post-challenge (**A, B, E**). The same statistical analysis was used to evaluate if there was a difference between the Benin 97/1 challenged Group A and Group C pigs (**C**). (**D**) Repeated measures two-way ANOVA, with Geisser-Greenhouse correction, and Tukey’s multiple comparisons test was used to evaluate differences between Group E and G, as there were no missing values (i.e. pigs culled before the 6th day). * represents P-values, where * <0.05, *** <0.001 and **** <0.0001.


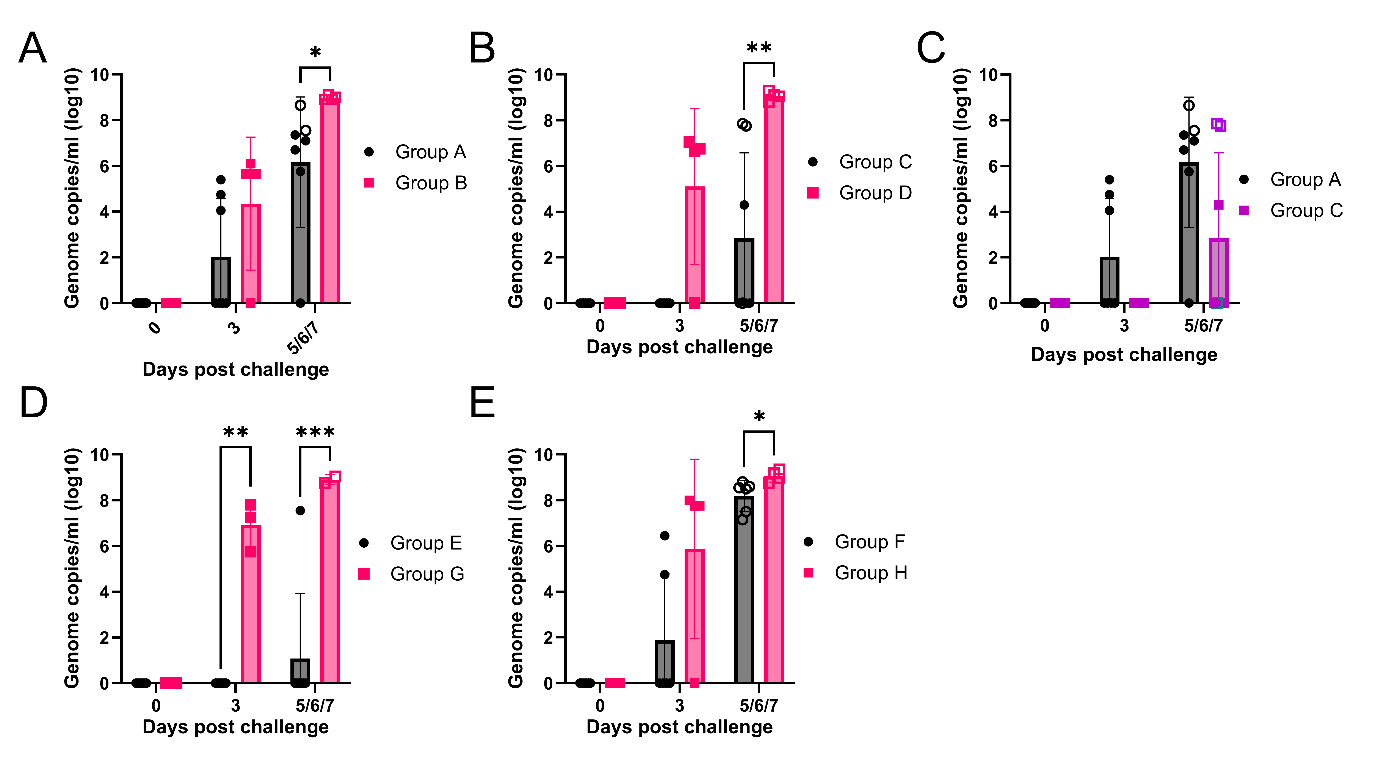


**Figure S3. Comparison of viral genome copies post-challenge.** Bar charts show the genome copies in a millilitre of blood in (**A**) Groups A and B, (**B**) Groups C and D, (**D**) Groups E and G, as well as (**E**) Groups F and H, on day 42 (0 days post-challenge), day 45 (3 days post-challenge and combined days 47 to 49 (5-, 6- and 7-days post-challenge). Open symbols (○ and □) represent pigs that were culled on that day at moderate humane endpoints. Repeated measures two-way ANOVA with Geisser-Greenhouse correction, and Tukey’s multiple comparisons test was performed to evaluate the differences between vaccinated-challenged and naïve-challenged groups over 6 days post-challenge (**A, B, E**). The same statistical analysis was used to evaluate if there was a difference between the Benin 97/1 challenged Group A and Group C pigs (**C**). (**D**) Two-way ANOVA, with Geisser-Greenhouse correction, and Tukey’s multiple comparisons test was used to evaluate differences between Group E and G. * represents P-values, where * <0.05, ** <0.01 and *** <0.001.

**
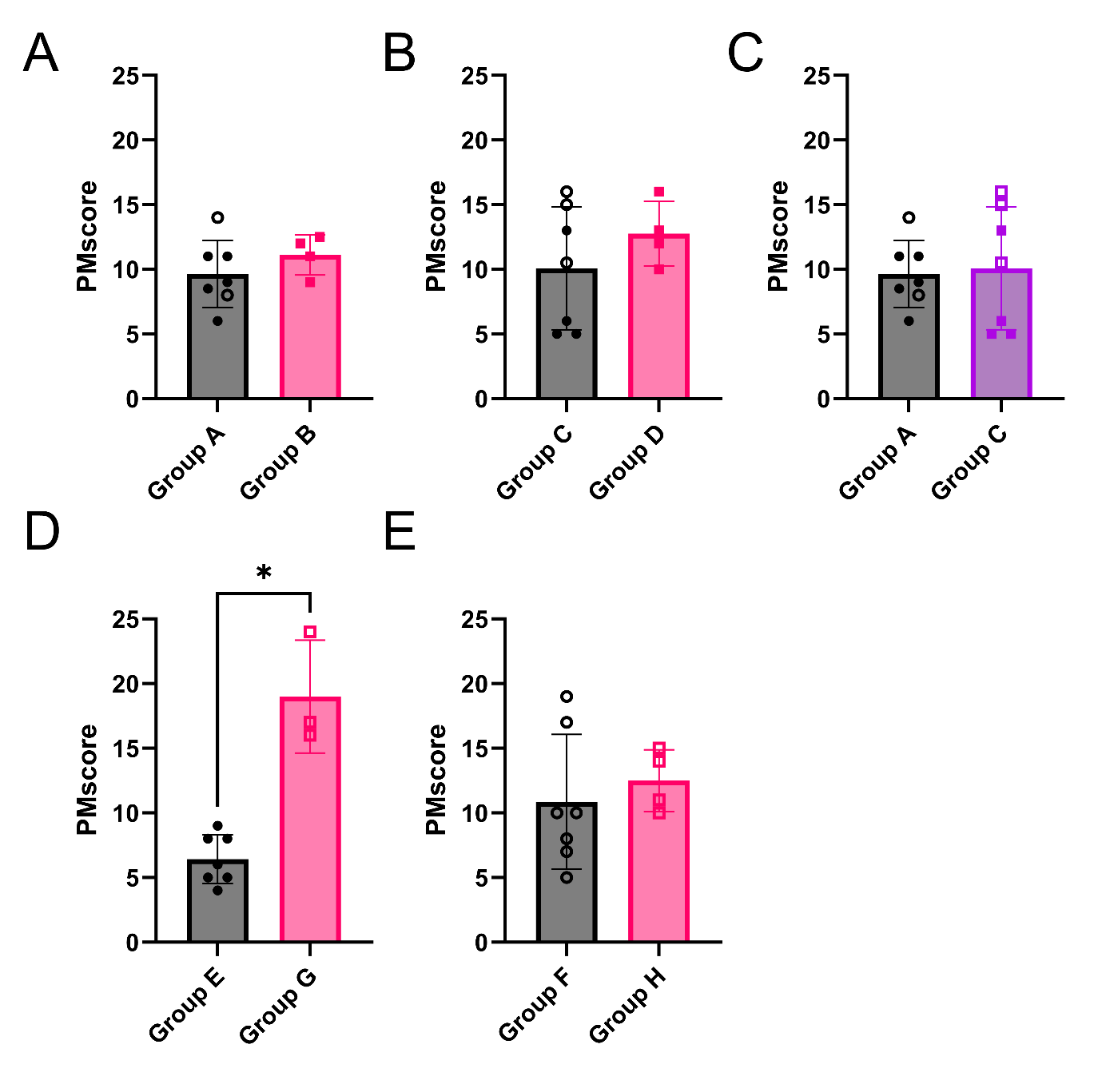
**

**Figure S4. Comparison of post-mortem scores.** Bar charts show the cumulative post-mortem scores between groups, where (**A**) shows Groups A and B, (**B**) Groups C and D, (**D**) Groups E and G and (**E**) Groups F and H during necropsy. Open symbols (○ and □) represent pigs that were culled at moderate humane endpoints. Unpaired, non-parametric, Mann-Whitney test, with two-tailed P value was used to compare the total cumulative score between the groups of pigs. * represents P-values, where * <0.05.

**
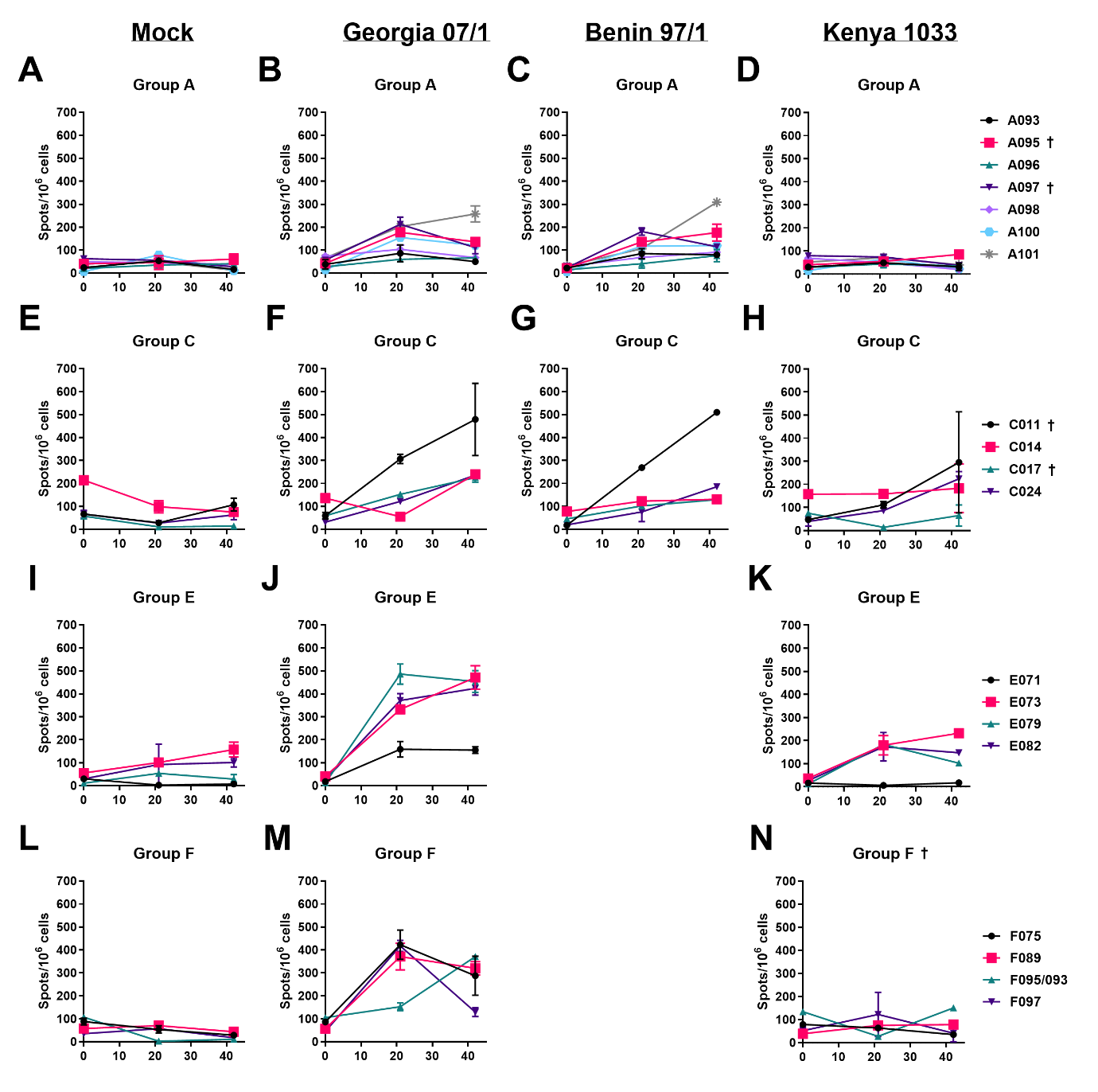
**

**Figure S5. Numbers of IFN-γ producing cells recorded for individual pigs following stimulation of PBMCs with different virus isolates or mock-stimulation.** PBMCs collected pre-immunisation, pre-boost or pre-challenge were stimulated with mock (**A, E, I, L**), ASFV genotype II, Georgia 2007/1 (**B, F, J, M**), ASFV genotype I, Benin 97/1 (**C, G**) and ASFV genotype IX, Kenya 1033 (**D, H, K, N**). The y-axis shows numbers of IFN-γ producing cells per million cells and x-axis shows the day post-immunisation. Results for individual pigs from each group are shown and the experimental group and pig number labelled on the graph. † denotes pigs that were culled at moderate humane endpoints after challenge. In Group F, F095/F093 is shown due to the culling of F095 after 7 dpi, and for the subsequent ELISpot assay at 21 and 42 dpi, PBMC from pig F093 was used.


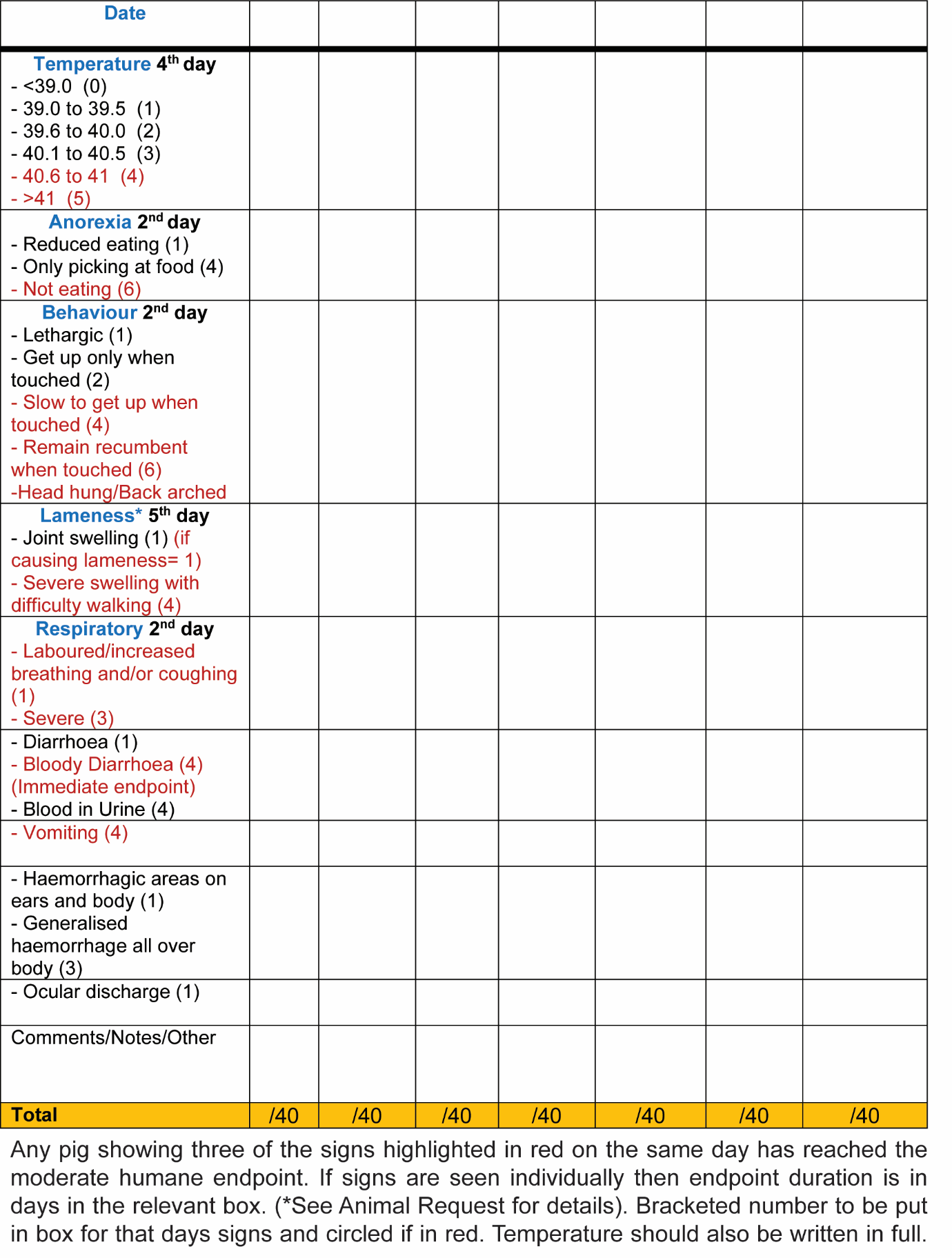


**Figure S6. Clinical scoring sheet.** Clinical signs are described in the left column with designated scores shown in brackets. Those signs contributing to the moderate severity humane end point are indicated in red. Daily scores are added in consecutive columns. The moderate severity humane endpoint is described in Supplementary Methods.

# Supplementary Methods

## In vivo immunisation and challenge

All experiments were conducted at the International Livestock Research Institute (ILRI), Kenya in licensed animal facilities. The studies at ILRI, Kenya were approved by the institutional animal care and usage committee reference numbers 2021-21, 2022-05 and 2022-33. A mix of female and castrated male Landrace x Duroc crosses were used. At ILRI, pigs were maintained in a quarantine facility before the start of the experiment and confirmed to be antibody and PCR negative for ASF. Animals were confirmed clinically healthy prior to the start of the study and were weighed and randomly allocated to groups based on body weights on Day -7. Clinical scoring was conducted daily [1] (Figure S2). Statistical analyses to evaluate the differences in temperatures and clinical scores between vaccinated and naïve pigs were conducted from 42 days post-immunisation (dpi) (0 days post-challenge (dpc)) up to 6 or 7 dpc. Two-way ANOVA either using the mixed effect model (when missing values are present) or repeated measures model, both with Geisser-Greenhouse correction was done alongside Tukey’s multiple comparisons test (GraphPad Prism 10). Blood samples were collected on different days post-immunisation and at termination to measure viremia and antibody responses. Animals were euthanised when they reached a pre-determined moderate severity humane endpoint (see description below) or at the end of the study. At necropsy, organs were observed for signs of macroscopic lesions typical of African swine fever virus infection. Scoring of lesions was carried out [2] and comparison between groups of pigs was done using the unpaired, non-parametric Mann-Whitney test (GraphPad Prism 10).

Figure 1 shows the schedule of 3 experiments conducted. In experiments 1 to 3, 4 groups of pigs (A, C, E and F) were immunised intramuscularly with GeorgiaΔDKE-Cmut. Viruses were back titrated to verify dosages used.

### Moderate severity humane endpoint

*Pyrexia.* On the third consecutive day of temperatures of 40.6°C or above the animal will be euthanised if it shows any of the clinical signs listed below that contribute towards an endpoint. For example, an animal that has had a temperature of 40.6°C or above for three consecutive days and was refusing food on the third day of temperature would be euthanised.

Any pig that has had four consecutive days of temperatures of 40.6°C or above will be euthanised regardless of any other clinical signs. The normal body temperature for pigs is 39°C but may vary between 38.0 and 39.9°C.

*Behaviour.* The animal will be euthanized if it is slow to get up when touched and/or refuses to get up when touched.

*Anorexia.* The animal will be euthanized if it refuses food for two consecutive days.

*Digestive system.* The animal will be euthanized if it has haemorrhagic diarrhoea.

*Vomiting.* The animal will be euthanized if vomiting is observed on the third day of pyrexia in association with two other of the above clinical signs.

*Respiratory system.* The pig will be euthanized if it shows laboured breathing for two consecutive days.

*Seizures.* Any pig suffering procedure related seizures will be euthanised immediately.

*Lameness attributed to a regulated procedure.* All animals showing lameness will be treated with antibiotics and /or suitable analgesia at first signs. Those animals showing non-weight bearing lameness will be euthanized 48 hours after treatment if they do not show improvement. Those animals showing weight bearing lameness will be euthanized at the beginning of the fifth consecutive day after treatment if lameness has not improved significantly. If there is a recurrence of the lameness, due to a regulated procedure, within 21 days from initial onset of lameness the animal will be euthanized on the same day. If the animal becomes lame, due to a regulated procedure, more than 21 days from the first signs of initial lameness it will be treated as if it was the first instance as point one above. If the animal becomes lame due to a regulated procedure a third time, it will be euthanized on the same day.

*Combinations of clinical signs.* Any animal showing three or more of the above clinical signs combined on a single day will be euthanized on the same day even if the duration of the individual endpoints has not been reached.

*Other.* If in any individual case it is considered necessary to maintain animals beyond these severity limits, the Home Office inspector will be consulted. If pigs develop clinical signs of disease which are thought to be due to an adventitious infection, appropriate treatment may be given on the advice of the duty veterinarian. If the animal does not respond to treatment, it will be killed on the advice of the named veterinary surgeon.

## Detection of ASFV genome in blood

DNA from whole blood was extracted using the Zymo Quick-DNA™ Miniprep kit according to the manufacturer's instructions. Extracted DNAs were amplified in duplicates for detection by PCR, which was performed using QuantStudio 5 system (Applied Biosystems, Waltham, MA, United States). Standard curves from an ASFV p72 for Kenya 1033 genotype IX mimic plasmid were included in every run to allow for genome quantification.

Two-way ANOVA either using the mixed effect model (when missing values are present) or repeated measure model, both with Geisser-Greenhouse correction was done alongside Tukey’s multiple comparisons test (GraphPad Prism 10) to evaluate differences in the levels of viral genome copies between the immunised and naïve pigs after challenge.

## ASFV VP72 antibody ELISA

A blocking ELISA against ASFV VP72 protein (INgezim PPA COMPAC, Ingenasa) was used to measure ASFV VP72 antibodies in serum collected from pigs at different times point throughout study as described by the manufacturer. Serum samples were first diluted in serum diluent buffer at 1:1 ratio. The diluted serum, negative and positive controls were added onto wells coated with inactivated VP72 antigen and incubated at 37°C for 1h. Following four washes with wash buffer, diluted VP72 specific monoclonal antibody -peroxidase conjugate was added. After incubation at 37°C for 30 min, the plates were washed five times with wash buffer. The binding of VP72 antibodies was detected via a colorimetric reaction after the addition of the TMB substrate and STOP solution. The optical density (OD) was then read at 450nm on a microplate reader BioTek with Gen5 software. The percentage of blocking was calculated using the following equation, ((negative control OD-sample OD))/(negative control OD-positive control OD) X 100%. Samples above 50% blocking were considered as positive, while anything below 40% was considered as negative. Samples with blocking between 40-50% are considered as doubtful.

## IFN-γ ELISpot assay to measure cellular responses in pigs

PBMCs were isolated from whole blood collected in EDTA vacutainers via Histopaque-1033. Isolated PBMCs were stimulated overnight with 10^5^ HAD_50_ ASFV, an equivalent volume of mock inoculum, or 20μg/mL phytohemagglutinin as a positive control. After lysis and incubation with biotinylated anti-porcine IFN-γ monoclonal antibody (Invitrogen) followed by streptavidin alkaline phosphatase (Caltag), the AP conjugate substrate kit (Bio-Rad) was used to develop spots. The spot forming cells (SFC) were counted using an ELISpot assay reader system (Immunospot, CTL).

# Supplementary References

1. King K, Chapman D, Argilaguet JM, et al. Protection of European domestic pigs from virulent African isolates of African swine fever virus by experimental immunisation. Vaccine. 2011 Jun 20;29(28):4593-4600.

2. Galindo-Cardiel I, Ballester M, Solanes D, et al. Standardization of pathological investigations in the framework of experimental ASFV infections. Virus research. 2013;173(1):180-190.
